# Lynch Syndrome—Impact of the Type of Deficient Mismatch Repair Gene Mutation on Diagnosis, Clinical Presentation, Surveillance and Therapeutic Approaches

**DOI:** 10.3390/medicina61010120

**Published:** 2025-01-14

**Authors:** Tudor Razvan Grigorie, Gheorghe Potlog, Sorin Tiberiu Alexandrescu

**Affiliations:** 1 Department of Surgery, Faculty of Medicine, Carol Davila University of Medicine and Pharmacy, 050474 Bucharest, Romania; mihai.grig@gmail.com; 2 Department of Hepato-Bilio-Pancreatic Surgery, Emergency University Hospital Bucharest, Splaiul Independentei 169, Sector 5, 050098 Bucharest, Romania; 3 Center for Digestive Diseases and Liver Transplantation, Fundeni Clinical Institute, 022328 Bucharest, Romania; g.potlog@yahoo.com

**Keywords:** Lynch syndrome, deficient mismatch repair gene, extended colectomy, surveillance, prophylactic colectomy, prophylactic total hysterectomy, bilateral salpingo-oophorectomy

## Abstract

In today’s world, with its continuing advancements in genetics, the identification of Lynch syndrome (LS) increasingly relies on sophisticated genetic testing techniques. Most guidelines recommend a tailored surveillance program, as well as personalized prophylactic and therapeutic approaches, according to the type of dMMR gene mutation. Carriers of path_MLH1 and path_MSH2 genes have a higher risk of developing colorectal cancer (CRC), despite intensive colonoscopic surveillance. Conversely, carriers of path_MSH6 and path_PMS2 genes have a lower risk of developing CRC, which may be due to their lower penetrance and later age of onset. Thus, carriers of path_MLH1 or path_MSH2 would theoretically derive greater benefits from total colectomy, compared to low-risk carriers (path_MSH6 and path_PMS2), in which colonoscopic surveillance might achieve an efficient prophylaxis. Furthermore, regarding the risk of endometrial/ovarian cancer development, there is a global agreement to offer both hysterectomy and bilateral salpingo-oophorectomy to path_MLH1, path_MSH2 and path_MSH6 carriers after the age of 40. In patients with CRC, preoperative knowledge of the diagnosis of LS is of tremendous importance, due to the high risk of metachronous CRC. However, this risk depends on the type of dMMR gene mutation. For carriers of the high-risk variants (MLH1, MSH2 and EPCAM) who have already developed colon cancer, it is strongly recommended a subtotal or total colectomy is performed, while partial colectomy followed by endoscopic surveillance is an appropriate management approach to treat colon cancer in carriers of the low-risk variants (MSH6 and PMS2). On the other hand, extended surgery for index rectal cancer (such as total proctocolectomy) is less effective than extended surgery for index colon cancer from the point of view of metachronous CRC risk reduction, and is associated with a decreased quality of life.

## 1. Introduction

The tremendous developments in understanding the molecular basis of cancers over the last decade allow for a refined prognostic estimation and personalized therapeutic approach in most oncologic patients [1,2,3,4]. Lynch syndrome (LS), also known as hereditary non-polyposis colorectal cancer (HNPCC), is an inherited genetic disorder that significantly increases an individual’s risk of developing various types of cancer, particularly colorectal cancer (CRC) and endometrial cancer (EC) [5]. It is caused by inherited mutations in one of the mismatch repair (MMR) genes (MLH1, MSH2, MSH6, or PMS2) or in the epithelial cell adhesion molecule (EPCAM), which leads to the epigenetic silencing of MSH2. The condition follows an autosomal dominant pattern of inheritance. First-degree relatives (parents, siblings and children) have a 50% chance of being affected by LS.

Individuals with LS have an increased lifetime risk of developing CRC (up to 80%) and EC (up to 60%), as well as other cancers, including ovarian (up to 15%), gastric (up to 18%), urinary tract (up to 20%), pancreatic (4%), small intestine cancers, glioblastoma (Turcot syndrome) and sebaceous neoplasms (Muir–Torre syndrome) [6,7,8,9,10,11,12].

LS is the most common hereditary form of CRC. It accounts for 2–4% of all CRC diagnoses [7,13]. In patients with LS, CRCs have an adenoma-carcinoma progression ratio of almost 1:1, with an estimated adenoma-to-cancer transformation time of 1–3 years. This contrasts with sporadic cases, which have a ratio of 30:1 and an estimated transformation time of 8–17 years. If left untreated, the majority of polyps will become malignant, with about 70% of patients developing cancer by age 70 and 80% by age 85. Additionally, there is a higher incidence of metachronous and synchronous colon cancers, with a second primary CRC occurring in up to 30% of patients within 10 years and 50% within 15 years [14].

LS carriers/patients have distinct clinic, evolutive and prognostic features, depending on the type of deficient MMR (dMMR) gene. In this narrative review, we present the clinical impact of each of these genes’ mutations, as well as the personalized therapeutic approach according to the type of genetic mutation that led to the development of LS. We also present the available data on the usefulness of screening and surveillance programs for patients with LS. Finally, we discuss prophylactic approaches that should be employed in case of each gene’s mutations.

## 2. The Clinical Impact of Distinct Genetic Mutations

LS is caused by germline mutations in DNA mismatch repair genes, including MLH1, MSH2, MSH6 and PMS2 [15,16]. Mutations in these genes lead to microsatellite instability, a hallmark of Lynch syndrome-associated tumors [17].

MLH1 and MSH2 are the most frequently mutated genes in patients with LS, accounting for approximately 70% of the identified mutations (32% in MLH1 and 38% in MSH2) [13,18]. The carriers of pathogenic variants in MLH1 and MSH2 genes have a significantly higher risk of developing CRC at a younger age compared to carriers of pathogenic variants in MSH6 or PMS2 genes [19]. Individuals with mutations in the MSH2 gene have a higher likelihood of developing extracolonic cancers and a lower frequency of CRC compared to those with mutations in the MLH1 gene [20,21]. MSH6 mutations are more commonly associated with gastrointestinal and endometrial cancers that typically occur at a later age [22,23].

Some studies have shown that constitutional 3′ deletions of EPCAM can lead to LS by causing epigenetic silencing of MSH2 in EPCAM-expressing tissues, which results in a tissue-specific deficiency of MSH2. Kempers et al. conducted a cohort study comparing 194 patients with an EPCAM deletion to 473 patients with mutations in MLH1, MSH2, MSH6, or a combined EPCAM-MSH2 deletion. The study found that carriers of an EPCAM deletion had a 75% cumulative risk of developing CRC before the age of 70, similar to that of carriers of combined EPCAM-MSH2 deletions or MSH2 mutations, but higher than that observed in MSH6 mutation carriers. However, only those with deletions extending near the MSH2 promoter showed an increased risk of endometrial cancer [24].

A change in any of the above-mentioned MMR genes can lead to the accumulation of numerous errors in the DNA repetitive sequences known as microsatellites, which occur throughout the genome. This process is known as microsatellite instability (MSI) and is present in LS, but not exclusive to it. Therefore, not all the patients with MSI have LS. To enhance the detection of individuals with LS, “universal tumor screening” is recommended. In this approach, all individuals newly diagnosed with CRC undergo either tumor-based dMMR genetic testing or immunohistochemistry (IHC) testing to check for the absence of DNA mismatch repair (MMR) proteins (MLH1, MSH2, MSH6 or PMS2). The latter method achieves a sensitivity of 100% (95% CI, 99.3–100%) and a specificity of 93.0% (95% CI, 92.0–93.7%) for identifying individuals with Lynch syndrome [25,26,27,28].

Traditionally, the diagnosis of LS typically began with clinical suspicion, particularly in individuals with a family history of CRC or other Lynch syndrome-associated cancers. The Amsterdam II criteria and the revised Bethesda guidelines were commonly used to identify individuals who may benefit from further genetic evaluation [29,30,31]. The shortcomings of using this strategy were, on one hand, that 50% of patients who met these criteria do not actually have LS and, on the other hand, that these criteria were missing in 50% of LS patients. For these reasons, testing for the MMR status of the tumors is nowadays recommended. If IHC staining for MLH1 (alone or with PMS2) is abnormal, testing for the BRAF mutation or MLH1-promoter methylation should be performed to detect tumors lacking DNA-MMR.

Somatic BRAF mutations occur in a small fraction of CRCs overall [32], but are present in 69% to 78% of CRCs with MLH1 promoter methylation. These mutations are virtually never seen in Lynch syndrome-associated cancers, making the presence of a BRAF mutation highly predictive of a sporadic origin and a high negative predictive value for LS [33,34]. If the test is negative, germline mutation testing for LS should be conducted. A multigene panel test is available, particularly for individuals diagnosed before the age of 50 [35,36].

In the absence of available tumor data or known mutations, online tools such as PREMM5 and MMR Predict help in estimating an individual’s risk of carrying an MMR mutation [6,37,38,39]. Given the complexities involved in selecting and interpreting the tests, as well as the potential implications of the results for the family, genetic counseling should precede and also succeed germline mutation testing.

## 3. Screening and Surveillance in Lynch Syndrome Patients

In LS patients, screening for CRC by colonoscopic surveillance has been generally accepted as a method for providing greater life expectancy. But the benefits offered by screening methods and surveillance are debatable for other cancers that put LS patients at risk.

Screening for CRC by colonoscopy is recommended for people at risk of (first-degree relatives who have not had genetic testing of known MMR gene mutation carriers) or with LS. Colonoscopies should be performed every 1 to 2 years, starting at age 25 or 5 years before the youngest case in family. Recent European guidelines from the EHTG and ESCP, based on PLSD studies (the prospective LS database), recommend a tailored surveillance program according to the type of dMMR gene mutation. Thus, for MLH1, MSH2 and MSH6, colonoscopic surveillance is recommended every 2 to 3 years, and for PMS2, surveillance every 5 years may be considered [5]. The fecal immunochemical test (FIT) is extensively utilized as a screening tool for CRC in the general population; however, its role in LS surveillance remains under investigation and is not yet well established. Recent studies have demonstrated that the FIT has low sensitivity (23%) for detecting adenomas. Although sensitivity for advanced adenomas reached 66.7%, the overall detection rates for adenomas are insufficient to replace colonoscopy as the primary surveillance method [40,41]. Nevertheless, the FIT may hold potential as an augmentative tool to complement colonoscopy in specific scenarios, warranting further investigation into its supplementary role and integration into LS surveillance strategies.

Although endometrial and ovarian cancer screening does not have proven benefits in women with LS according to some studies [38], more recent data suggest that yearly gynecological examination, pelvic ultrasound, CA125 and endometrial biopsy from age 30 to 35 may be useful [39,42,43,44,45,46]. In regions with a high incidence of gastric cancer and in families with a history of gastric neoplasms, upper endoscopy surveillance may be recommended every 2–4 years, with gastric biopsying of the antrum starting at the age of 30–40 years [47,48,49]. For the urinary tract, no consensus currently exists regarding the proper screening protocol, and great variability still exists regarding the methods and the starting age of screening, ranging from 25 to 50 years [50]. Annual magnetic resonance imaging (MRI) and/or endoscopic ultrasound (EUS) surveillance may be considered for individuals with LS who have one first-degree relative affected by pancreatic cancer, although additional supporting evidence is needed to back up this recommendation [51]. Routine screening for prostate and breast cancer is not recommended beyond what is advised for the general population. A skin exam every 1 to 2 years with a healthcare provider experienced in recognizing Lynch syndrome-associated skin manifestations is recommended. The optimal age to begin surveillance is uncertain and can be individualized based on personal and family history [39].

Recent observations suggest that future knowledge about the changes of gut microbiota in LS may be a useful tool for the surveillance of these patients. Research over the last few years suggests that the gut microbiota may have a different pattern in LS and non-LS patients, probably due to the underlying differences in epithelial biology and immunology [52,53,54]. For example, Rifkin et al. showed that Veillonella was enriched and Faecalibacterium and Romboutsia were depleted in LS [52], whereas Mori et al. suggested that microbiota pattern associated with LS is characterized by an over-representation of Faecalibacterium prausnitzii, Parabacteroides distasonis, Ruminococcus bromii, Bacteroides plebeius, Bacteroides fragilis and Bacteroides uniformis species [53]. The interaction between the specific fecal microbiota pattern and the altered immune surveillance of LS patients may play a critical role in CRC development [55]. Thus, Yan et al. found that a subset of Clostridiaceae was depleted in stool biopsies, corresponding with baseline adenomas, while Desulfovibrio was enriched both in stool and in mucosal biopsies [54]. Their observations suggest that although prospective monitoring of microbiome has limited benefit in the early detection of adenoma, these early changes in microbiota may play a causal role in colonic neoplasia [4]. Moreover, Mori et al. suggested that despite the possible existence of a fecal microbiota pattern associated with a LS genetic background, there were no differences between microbial communities of patients with LS and CRC, and those observed in patients with LS and gynecologic malignancies [53]. However, future studies are needed to better understand the relationship between microbiota and cancer development in LS patients, and how the changes in microbiota can be used in the early detection of Lynch syndrome-related malignancies. Furthermore, the adequate manipulation of microbiota could represent a future therapeutic option to avoid the development of some malignancies related with LS.

## 4. Preventive Measures for Lynch Syndrome

Aspirin may be considered as a preventive measure against cancer in individuals with LS, although the optimal dosage remains unclear. The Colorectal Adenoma/Carcinoma Prevention Programme 2 (CAPP2) demonstrated a 60% reduction in the incidence of CRC and other Lynch syndrome-associated tumors in individuals who took 600 mg of aspirin daily for at least two years, compared to those who received a placebo [56]. The ongoing CAPP3 study aims to determine the most effective dose by comparing daily aspirin intake at 600, 300 and 100 mg. The European guidelines from the European Hereditary Tumour Group (EHTG) and the European Society of Coloproctology (ESCP) recommend the acetylsalicylic acid dose should be a minimum of 75–100 mg daily and this dose should be increased for people with above-average body mass [5]. However, the American College of Gastroenterology does not recommend the routine use of aspirin for the chemoprevention in LS [57].

In certain cases, prophylactic surgical interventions such as total colectomy or risk-reducing salpingo-oophorectomy may be considered for individuals with LS who are at particularly high risk of developing certain cancers.

### 4.1. Prophylactic Surgery for CRC in Patients with LS

Prophylactic surgery aims to remove organs before cancer develops, reducing the potential risk. The decision regarding which operation is preferable should be made on the basis of individual patient’s factors and preferences, with special emphasis on the risk of metachronous CRC, the functional consequences of surgery, the patient’s age and the commitment of the patient to continue colonoscopic surveillance [58]. The term of prophylactic total colectomy is defined either by total colectomy with ileo-rectal anastomosis, or proctocolectomy ended with ileal-anal pouch anastomosis (IPAA) or with ileostomy. In contrast to FAP, in which proctolectomy is the procedure of choice, Syngal et al. [59] showed that minimal benefit is derived from performing proctocolectomy rather than subtotal colectomy on patients with LS. By contrary, Henegan et al. [60] consider that a true prophylactic surgery is total proctocolectomy with IPAA or with end ileostomy because we can no longer talk about prophylaxis in rectal sparing surgery as it leaves the rectum in the LS patient, who then has a 1% per year risk of developing metachronous rectal cancer for the first 12 years [61]. However, patients with the rectum left in place could be regularly surveilled via a rectoscopy, which is more easily performed and accepted by the patient than a full colonoscopy.

The timing for surgery should be evaluated on an individual basis, taking into consideration gender, familial pattern of cancer and the age when cancers occur in relatives. Prophylactic surgery needs to be performed at an earlier age than the age of cancer occurrence in the youngest relative [61]. Prophylactic colectomy requires the careful evaluation of its implications, as it can significantly impact quality of life, lead to considerable morbidity and carries mortality risks. Individuals with LS have a lifetime colorectal cancer (CRC) risk of about 70%, indicating that nearly 30% out of these surgeries may be unnecessary for patients that would never develop CRC. Moreover, some of patients could eventually develop types of cancer other than CRC, and the prophylactic colectomy would not only be futile, but could also worsen their quality of life [62].

Llach et al. considered that prophylactic colectomy or proctocolectomy in healthy LS patients is not indicated due to the efficacy of colonoscopy on CRC mortality reduction, but they argue that there may be a role for prophylactic colorectal surgery in the secondary prevention of CRC [57,63,64,65].

Prophylactic proctocolectomy is recommended for patients with a pathologic germline mutation in the APC gene leading to familial adenomatous polyposis (FAP) [66]. Similarly, risk stratification by affected MMR gene may help identify the LS patients more prone to developing CRC. Thus, carriers of path_MLH1 or path_MSH2, who have a higher risk of developing CRC, would theoretically derive a greater benefit from total colectomy compared to carriers of the low-risk variants (path_MSH6 and path_PMS2), in which colonoscopic surveillance might achieve an efficient prophylaxis (Table 1).

Prophylactic colorectal surgery might be considered in some particular situations, e.g., for mutation carriers who are unable to undergo surveillance, for patients who are noncompliant with surveillance examinations or have endoscopically unresectable adenomas with severe dysplasia, or for patients with severe distress regarding the development CRC who prefer surgery to surveillance [63,66].

Further prospective studies are necessary in order to elaborate clear guidelines concerning the role of prophylactic CRC surgery for patients with LS before developing CRC.

### 4.2. Prophylactic Surgery for Gynecologic Cancers in Lynch Syndrome

Women with LS have a 40 to 60% lifetime risk of EC and a 10 to 12% lifetime risk of ovarian cancer [62].

Therefore, for women with LS, prophylactic hysterectomy and bilateral salpingo-oophorectomy (BSO) can, at least theoretically, significantly reduce the risk of endometrial and ovarian cancers. Such surgical interventions may provide a substantial risk reduction for gynecological cancers, which are common in LS, and may be an important consideration for women with this condition, particularly those who have completed childbearing (>40 years) [8,64,67,68].

Women who carry an MSH2, MLH1 or MSH6 germline mutation and who present with CRC in the absence of distant metastases will present an extraordinarily high lifetime risk for carcinoma of the endometrium and/or ovary, therefore prophylactic hysterectomy and oophorectomy may also be considered for female patients with LS [61,69]. These procedures aim to reduce the risk of gynecologic cancers in this high-risk population.

Several surgical techniques have been proposed and implemented to achieve this goal. The most common surgical techniques include prophylactic total hysterectomy and bilateral salpingo-oophorectomy (THBSO). The opportunity for combining THBSO with colectomy should be discussed with the patient, taking into account the patient’s age, comorbid conditions, plans for fertility and specific family history of cancer.

Although studies conducted before 2006 showed uncertain benefits in reducing gynecologic cancer risk after prophylactic surgery, the benefit of prophylactic THBSO was clearly demonstrated in a case-matched study reported by Schmeler et al. [67].

The timing of surgery should be individualized based on comorbidities, family history, LS gene and whether childbearing is complete [70].

In 2021, the ESGO-ESTRO-ESP consensus recommended surveillance for endometrial cancer in patients with LS starting at the age of 35 by annual transvaginal ultrasound (TVUS) and annual or biennial endometrial biopsy. Prophylactic THBSO should be considered at the end of childbearing and preferably before 40 [71], or even sooner if the patient does not wish to preserve fertility. The lack of consensus and evidence for the effectiveness of surveillance may also be used to enhance the argument for prophylactic THBSO when the opportunity arises, either at the time of prophylactic or curative colectomy in women with LS or as a separate operation once childbearing is complete for patients wanting to preserve fertility [62,72].

In a 2020 survey study that involved 18 countries, there was global agreement (>90%) in favor of offering both hysterectomy and BSO to carriers of path_MLH1, path_MSH2 and path_MSH6 genes after the age of 40 [73] (Table 1).

Because there is a wide variation in how, when and to whom risk-reducing gynecological surgery is offered, there is a clear need for further research in the field of care for the management of gynecological cancer risk.

## 5. Curative Surgery for Colorectal Cancer in Lynch Syndrome

The primary focus for individuals with LS is to prevent and/or detect cancer at an early stage. This involves using pre-symptomatic screening methods and opting for surgical removal when feasible.

Colorectal resection surgery on patients with Lynch syndrome who have been diagnosed with colorectal cancer should adhere to at least the same oncological standards used for those with non-hereditary colorectal cancer. It is crucial to ensure that these patients receive comprehensive and personalized care, considering the unique aspects of LS.

Moreover, a multidisciplinary approach involving a team of experts, including colorectal surgeons, gastroenterologists, oncologists and genetic counselors is fundamental for optimizing the surgical outcomes and long-term prognosis of these patients.

Additionally, post-operative surveillance is of utmost importance to facilitate early detection of any possible recurrence or the development of secondary malignancies.

With advances in medical research, targeted therapies and immunotherapies are emerging as potential treatment options for patients with CRC associated with Lynch syndrome, further emphasizing the need for individualized treatment plans.

### 5.1. Surgical Management of Primary Colon Cancer in Lynch Syndrome

Individuals with LS face a significant risk of developing life-threatening colorectal and endometrial cancers, with incidences reaching 40–80% by the age of 75 [74]. Despite surveillance efforts, the effectiveness of early detection remains limited, mainly because accelerated adenoma-to-carcinoma progression has been reported in patients with LS, with estimated polyp-to-cancer dwell times of 35 months compared with 10 to 15 years in sporadic cancer [75,76]. The limited effectiveness of colonoscopy can be explained by missed lesions on exploration, fast progression of newly formed adenomas, the fact that not all CRC in LS follow an adenoma-carcinoma pathway and the occurrence of induced lesions by multiple colonoscopies in MMR carriers [77]. Carriers of path_MLH1 and path_MSH2 genes have a higher risk of developing colorectal cancer, despite intensive surveillance colonoscopy [8,77,78,79]. Conversely, carriers of path_MSH6 [8,79] and path_PMS2 [80] genes have a lower risk of developing CRC, which may be due to their lower penetrance and later age of onset, and can be further reduced by regular colonoscopic surveillance or even become near to zero in carriers of PMS2 [78,81,82]. Characteristically, in LS, CRC develops at an early age, with right-sided tumor predominance (60–65%), along with extracolonic tumors of the endometrium, ovary, stomach, renal pelvis, ureter and other organs [62,74].

The surgical principles required when considering a case with CRC in the setting of LS should respect the following desideration: (1) the appropriate treatment of the primary tumor according to oncological principles applied in sporadic cases; (2) consideration of further risk reduction with prophylactic removal of larger parts of the non-neoplastic colon; (3) decrease morbidity and increase quality of life after colectomy [1]; (4) patient gender, age and general status; and (5) patient choice. In order to respect these principles, the range of surgical removal extends from limited resection/segmental colectomy, towards total colectomy with ileo-rectal anastomosis and finally to proctocolectomy completed with an IPAA or with end ileostomy. The extent of colorectal resection should be thoroughly discussed with the patient and the decision on this issue should consider patient’s gender, age, general status, willingness to adhere to the program of colonoscopic surveillance and the degree of distress regarding the development of metachronous CRC.

To avoid the misleading interpretation of the terminology used for surgical approaches, we will further define the terms “segmental colectomy” and “extended colectomy”. Segmental colectomy includes right or left hemicolectomy (for right or left colon cancer, respectively), segmental colectomy (for transverse colic cancers), and anterior resection or abdominoperineal resection (for rectal cancer). Extended colectomy includes extended right hemicolectomy, subtotal colectomy or total colectomy with ileo-rectal anastomosis (for colon cancer), and total proctocolectomy ended with IPAA or with end ileostomy (for rectal cancer).

When feasible, a minimally invasive approach (MIS) should be favored for patients with LS. Overall, the implementation of a MIS for patients with LS is highly recommended, as it optimizes surgical outcomes while prioritizing patient safety and well-being [83]. In the context of advancements in CRC surgery for patients with LS, the utilization of laparoscopic and robotic techniques has shown promising results in terms of reducing postoperative complications and improving recovery times [84].

#### 5.1.1. Segmental Colectomy for Index Colonic Cancer

The selection of the suitable surgical procedure should be made after carefully considering the patient’s unique factors and preferences. It is crucial that several aspects are taken into account, such as the risk of developing metachronous CRC, the age of the patient, the pathologic gene that determined LS and readiness to undergo surveillance colonoscopy. By thoroughly analyzing these factors, one can determine the most appropriate surgical procedure that will ensure optimal outcomes for the patient.

However, the vast majority of primary CRCs in LS patients are managed with segmental colectomy, simply because of the lack of preoperative recognition of the syndrome [62]. Some patients are susceptible to LS based on family history, but this can often be incomplete. Moreover, a majority of the patients with an unknown family history will be diagnosed by genetic testing of the colorectal specimen only after surgical removal.

Therefore, at present, it is strongly recommended that an immunohistochemical (IHC) evaluation of MMR genes expression is performed on the specimen attained by colonoscopic biopsy. If IHC staining revealed MSI-high status, genetic testing for germline mutations of MMR genes should be performed, in order to have a precise diagnosis before a surgical intervention.

Preoperative knowledge of the diagnosis of LS is of tremendous importance, due to the high risk of metachronous CRC in these patients. Thus, Parry et al. reveal that the risk of metachronous CRC is significantly reduced by 31% for every 10 cm of bowel removed, and Kim et al. report that a bowel resection of 25 cm or longer decreases the risk, as compared to less extensive resections [85,86]. Therefore, extended colectomy or even total/near total colectomy should be advocated in these patients.

Furthermore, every genetic variation in the MMR genes linked to LS (MLH1, MSH2, MSH6, PMS2 and EPCAM) carries a distinct risk of developing metachronous cancer. As a result, current guidelines distinguish between these genes when making recommendations for extension of colonic resection and surveillance programs. MLH1, MSH2 and EPCAM are classified as high-risk variants, and MSH6 and PMS2 as low-risk variants [5,20,74].

The latest NCCN version on Genetic/Familial High-Risk Assessment (version 1.2024—9 Spetember 2024 https://www.nccn.org/guidelines/guidelines-detail?category=2&id=1544) [39] predicted an up to 43% cumulative lifetime risk of metachronous CRC for MLH1 and MSH2 carriers who have segmental resection, and a lower risk for MSH6 carriers. For this reason, it is strongly recommended that a subtotal or total colectomy is performed for carriers of the high-risk variants who have developed CRC. There are limited data on PMS2, but no marked increase in risk for metachronous CRC has been reported in the available literature. Eikenboom et al. show that the risk of metachronous colorectal cancer did not differ between carriers of low-risk variants who had segmental colectomy and those of high-risk variants who had extensive colectomy, and they conclude that a partial colectomy followed by endoscopic surveillance is an appropriate management approach to treat colorectal cancer in carriers of low-risk Lynch syndrome variants [74] (Table 2).

Therefore, the decision to perform segmental versus total/near total colectomy should balance the risks of metachronous cancer according to the pathologic gene, the functional consequences of surgery and the patient’s age and wishes.

Compared to young and fit patients, elderly and frail patients are more susceptible to experience adverse outcomes following surgery. Such outcomes include postoperative complications, functional decline and worse quality of life after surgery. Advanced age in LS typically refers to patients over 60–65 years of age [8,87]. Although the Mallorca Group Surgery [88] recommends total abdominal colectomy with ileo-rectal anastomosis regardless of patient age, older patients have a relatively short life expectancy in comparison to younger patients, and quality of life means more than longevity for many of these patients [89].

Quality of life encompasses various aspects such as physical function, psychological well-being and social interactions. Surgery, especially in older, frail patients, can disrupt these areas and lead to a decrease in overall quality of life. In light of these challenges, it is important for healthcare professionals to carefully assess and manage the risks associated with surgery in older CRC patients. This includes implementing tailored approaches to optimize outcomes and minimize potential complications. By taking a comprehensive and individualized approach, healthcare teams can strive to improve the overall prognosis and postoperative experience for older LS patients with CRC [90,91]. For these frequently frail patients, it seems reasonable to perform a segmental colectomy instead of an extended colectomy, in order to minimize the postoperative morbidity and offer a better quality of life.

#### 5.1.2. Extended Colectomy for Index Colonic Cancer

Theoretically, extended colectomy reduces the amount of future colorectal tissue exposed to carcinogenesis, thus reducing the recurrence of CRC. The more extended the CRC resection, the lower the risk of metachronous CRC development. Subtotal colectomy with ileo-sigmoidostomy or total colectomy with ileo-rectal anastomosis significantly lowers the risk of developing future CRC, but does not eliminate it completely. However, surveillance is easier in these patients, as there is only a small portion of rectum (and sigmoid) that has to be monitored regularly by recto-sigmoidoscopy, and this investigation is better tolerated by patients. Although the recurrence rate is higher in patients treated by limited resection versus extended colonic resection (the rates of recurrent CRC after 10 years were 16% vs. 4%, respectively), the overall survival benefit of extended resection was not demonstrated by Natarjan et al. [92], probably due to the relatively small number of available subjects. However, de Vos tot Nederveen Cappel and colleagues show that life expectancy increased up to 2.3 years for patients who underwent extended colectomy at a younger age (under 47 years) compared to their counterparts treated with segmental resection [87].

Furthermore, Natarajan et al. recommend extended colectomy due to the increased incidence of metachronous CRC and the frequent necessity for a second abdominal surgery on patients who undergo limited resection [92].

The advantage of extended colectomy may be influenced by the age at first CRC. A decision analysis model pointed out that subtotal colectomy performed at 25 years of age in LS patients with CRC led to the greatest life expectancy [8].

Extended colectomy is also indicated in recurrent CRC because it is cost effective, and is favored by patients because it spares them from repeated colonoscopies and laparotomies [62,93]. As already mentioned, the EHTG and ESCP guidelines recommend extended colectomy (either total or subtotal colectomy ended with ileo-rectal anastomosis or with ileo-sigmoidostomy) for high-risk patients (path_MLH1 and path_MSH2 carriers) [5] (Table 2).

Thus, the extent of colonic resection in individuals with LS remains a complex and nuanced topic. While the fundamental principles of oncologic colorectal surgery apply, the unique considerations of this high-risk population must be carefully weighed. A tailored surgical approach based on tumor characteristics, gene mutations and patient’s risk factors and desires, as well as the potential for neoadjuvant therapy and organ-preserving strategies for patients with rectal cancer may optimize both oncologic and functional outcomes for individuals with LS [94,95].

Despite active surveillance in LS, more frequent colonoscopic surveillance did not reduce the incidence of metachronous CRC or stage at detection [96]. This is another reason for opting for extended CRC surgery in LS, even at the time of metachronous CRC. Nevertheless, many guidelines recommend colonoscopic surveillance every 1–2 years in LS patients [5,25,83].

As described above, the choice between segmental and extended colectomy for patients with colon cancer in LS involves weighing the benefits of reduced metachronous CRC risk against the potential for worse bowel function and lower quality of life [97].

### 5.2. Surgical Management of Primary Rectal Cancer in Lynch Syndrome

Although 60% of CRCs in Lynch syndrome occur on the right colon, about 10–15% of LS patients present with rectal cancer as an index tumor [98,99]. It is associated mostly with mutations in the MSH2 and MSH6 genes that are also present in extracolonic malignancies [100].

Some authors suggest that rectal cancer should be managed in the usual way, based on standard oncologic principles for sporadic rectal cancer, without a requirement for LS-specific approaches [63]. Thus, different guidelines recommend segmental resection (either anterior resection or abdominoperineal resection) in LS patients presented with index rectal cancer [5,101]. On the other hand, You et al. [102] consider that in dMMR genes carriers with rectal cancer, the surgical strategy should be tailored by addressing not only the rectal cancer (loco-regional control, distant metastases control and functional outcome) but also the issues associated with LS—the risk of metachronous CRC and the risk of cancer occurrence in other organs (especially ovarian or endometrial).

Thus, the surgeon should decide whether to perform a standard rectal resection according to the location of the primary tumor in the rectum or extend the resection to the remaining colon in order to reduce the risk of metachronous CRC (performing a total proctocolectomy ended with IPAA or an end ileostomy). The choice of localized vs. extended resection should be discussed with the patient and explained thoroughly, given the issues of regular colonoscopic surveillance, the risk of missed lesions at colonoscopy (in case of a limited resection) and the decreased quality of life and higher morbidity rates (observed after extended resections). Moreover, surgeons should discuss the possibility of performing prophylactic THBSO at the same time as the CRC surgery, given the increased risk of uterine and ovarian cancer in women with LS.

A recent study that compared the risk of metachronous CRC after surgical resection in two groups [colonic cancer (CC) and rectal cancer (RC) index group] found that the incidence of metachronous CRC was lower in the rectal group [103]. Another finding was that cause of death was associated with extracolonic LS tumors (mainly gynecologic in women) without any deaths due to CRC in the RC group, whereas in the CC group, 28.6% of deaths were associated with metachronous CRC. Therefore, considering the above-mentioned results, extended surgery for index rectal cancer (such as total proctocolectomy) is less effective than extended surgery in index colon cancer group, from the point of view of metachronous CRC risk reduction, and is associated with a decreased quality of life (Table 2). Also, prophylactic THBSO at the time of index surgery seem to be lifesaving in both groups. Although Kalady et al. and Win et al. propose total proctocolectomy with IPAA as a treatment of index rectal cancer, Chikatani et al. consider that this extensive surgery cannot be recommended as a standard treatment [103,104,105].

Since the published results are contradictory, further prospective studies are needed, with larger cohorts, in order to achieve definitive conclusions.

Other issues concerning the treatment of rectal cancer in patients with LS are the multimodal treatment and alternative approaches to TME.

The standard treatment of locally advanced (stage II and III) rectal cancer is preoperative (chemo)radiotherapy followed by surgery and adjuvant systemic chemotherapy [106,107]. The question of whether pelvic radiation can be skipped for certain patients is really important for LS patients with rectal cancer, mainly in sphincter-saving procedures in which radiation therapy may led to bowel dysfunction. Another aspect in LS patients is that they are generally young and the patient should be informed about the risks related to long term consequences of pelvic radiation: sexual dysfunction, hip fracture and fibrosis [108,109].

Regarding the surgical approach, radical total mesorectal excision (TME) is the mainstay treatment for patients with rectal cancer. Although laparoscopic, robotic and transanal-TME (TaTME) approaches improved the surgical armamentarium in TME, there is significant debate regarding the approach that achieves the best oncologic results [110].

Even though TME is the procedure of choice for patients with resectable rectal cancer without metastases and local excision (LE) techniques have been associated with inferior oncologic outcomes, in select cases, LE may be a recommended surgical alternative, due to the lower morbidity rates and better quality of life for select patients [110,111,112,113]. This could be particularly useful when treating elderly LS patients with multiple prior surgical interventions, or those who refuse stoma formation or an extended resection that can lead to bowel and functional disturbances [102].

Only 4% [114] of LS patients present with stage IV rectal cancer. In patients with unresectable metastases, the role of surgery is minimal and patients could benefit from systemic therapy, although up to 40% of these patients could be rendered to resectability [115,116]. Nevertheless, after curative intent treatment, such patients could achieve 5-year overall survival and disease-free survival rates up to 60% and up to 40%, respectively [94,117,118].

## 6. Conclusions

As the field is ever-evolving, it is crucial for clinicians to stay informed about the latest guidelines and recommendations regarding the management of LS.

Presently, the diagnosis of LS is genetic. The pathologic MMR gene has a huge impact on clinical presentation, on the risk of developing different types of cancers and, consequently, on the surveillance programs, prophylactic approaches and extent of colorectal resection. Pathologic variants of MLH1 or MSH2 genes are associated with a significantly higher risk of developing CRC and metachronous CRC after the resection of the index colon cancer. For these reasons, carriers of these high-risk variants would derive a greater benefit from total colectomy compared to carriers of the low-risk genes. Total or subtotal colectomies are recommended for treating index colon cancer in such patients. By contrary, extended surgery for index rectal cancer seems to be less effective than extended surgery in the index colon cancer group, from the point of view of metachronous CRC risk reduction. Furthermore, the performance of a total proctocolectomy (ended with either IPAA or an end ileostomy) is associated with a decreased quality of life and, for these reasons, this extensive surgery cannot be currently recommended as standard treatment. Prophylactic THBSO should be considered at the end of the childbearing age and preferably before 40 years of age or sooner if the patients do not wish to preserve fertility, especially in path_MLH1, path_MSH2 and path_MSH6 carriers.

Future studies should focus on refining the criteria for surveillance and intervention, and ensuring that patients receive individualized care based on their unique genetic profiles and personal circumstances.

## Figures and Tables

**Table 1:** Impact of genetic mutations on the risk of colon cancer development and prophylactic surgery (+++ high risk/strong recommendation, + low risk/weak recommendation).

Genes	Risk of Colon Cancer	Prophylactic Colectomy	Prophylactic THBSO
MLH 1	+++	+++	+++
MSH 2	+++	+++	+++
MSH 6	+	+	+++
PMS 2	+	+	+

**Table 2:** Extent of colorectal resection according to the index colorectal tumor location and genetic mutations (APR = abdominoperineal resection).

Index Tumor	MLH1	MSH2	MSH6	PMS2
Primary Colon Cancer	Total/subtotal colectomy	Total/subtotal colectomy	Segmental colectomy	Segmental colectomy
Primary Rectal Cancer	Anterior resection/APR	Anterior resection/APR	Anterior resection/APR	Anterior resection/APR

## Data Availability

No new data were created or analyzed in this study. Data sharing is not applicable to this article.
